# The stigmatization of patients with chronic pain due to assessed exaggeration of symptoms

**DOI:** 10.1080/24740527.2025.2583909

**Published:** 2025-12-15

**Authors:** Hance Clarke, Kenneth Craig, Rodrigo Deamo Assis, Nimish Mittal, Mary-Ann Fitzcharles

**Affiliations:** a Department of Anesthesia and Pain Management, Pain Research Unit, Toronto General Hospital, Toronto, Canada; b Transitional Pain Service, Toronto General Hospital, Toronto, Canada; c Professor Emeritus in the Department of Psychology, University of British Columbia, Vancouver, Canada; d Physical Medicine and Rehabilitations, Centre Intégré de Santé et Services Sociaux de l’Abitibi-Témiscamingue, Rouyn-Noranda, Canada; e Department of Anesthesia and Pain Management, University of Toronto, Toronto, Canada; f Temerty Faculty of Medicine, Division of Physical Medicine and Rehabilitation, University of Toronto, Toronto, Canada; g Department of Rheumatology, McGill University, Montreal, Canada; h Alan Edwards Pain Management Unit, McGill University, Montreal, Canada

**Keywords:** Chronic pain, exaggeration, stigmatization

## Abstract

Patients with chronic pain that cannot be explained by tissue abnormality may be accused of symptom amplification and at worst malingering. This is particularly relevant in the medicolegal setting where legal decisions are highly dependent on objective and validated information, conditions mostly lacking in the setting of chronic pain. When evaluations are conducted by assessors less familiar with current knowledge of pain mechanisms, subjective complaints of pain and associated symptoms such as fatigue and cognitive difficulties, are at risk of being misinterpreted leading to bias and stigmatization. In this commentary we will highlight some of the pitfalls that erroneously lead to a biased assessment of pain severity including failure to pay attention to psychological state and sociocultural influences, application of poorly reliable physical maneuvers, and use of neurocognitive testing of intentional cognitive dysfunction as a surrogate for dishonesty in pain and functional impairment report. Concerns about misinterpretation of exaggeration in persons with chronic pain are highlighted by recent report of symptom exaggeration in up to two thirds of those attending for an independent medical evaluation. Directives to help the medical assessor to provide pertinent information that will assist the courts in reaching a fair decision are discussed, with emphasis on need for a comprehensive assessment of biosocial factors, contextual variables and nonphysical evidence.

## RÉSUMÉ

Les patients souffrant de douleur chronique qu’on ne peut expliquer par une anomalie tissulaire peuvent être accusés d’amplifier leurs symptômes et, au pire, de simulation. Cela est particulièrement pertinent dans un contexte médico-légal où les décisions juridiques dépendent fortement de renseignements objectifs et validés, des paramètres le plus souvent absents dans les cas de douleur chronique. Lorsque les évaluations sont menées par des évaluateurs moins familiers avec les connaissances actuelles en matière de mécanismes de la douleur, les plaintes subjectives de douleur et les symptômes associés tels que la fatigue et les difficultés cognitives peuvent être mal interprétés, ce qui conduit à des préjugés et à la stigmatisation. Dans ce commentaire, nous mettrons en évidence certains écueils susceptibles de conduire à tort à une évaluation biaisée de la gravité de la douleur, notamment l’absence de prise en compte de l’état psychologique et des influences socioculturelles, l’application de manœuvres physiques peu fiables, et l’utilisation de tests neurocognitifs destinés à détecter un dysfonctionnement cognitif intentionnel, utilisés comme substituts pour inférer une malhonnêteté dans le rapportage de la douleur et de l’atteinte fonctionnelle. Des données récentes indiquent que jusqu’à deux tiers des personnes se présentant à une évaluation médicale indépendante voient leurs symptômes considérés comme exagérés, ce qui souligne l’importance du problème. Nous abordons également les directives visant à aider l’évaluateur médical à fournir des renseignements pertinents qui permettront aux tribunaux de prendre une décision équitable, en insistant sur l’importance d’une évaluation complète des facteurs biosociaux, des variables contextuelles et des éléments de preuve non physiques.

Two decades ago Craig and Badali introduced a special series on pain deception and malingering in patients with chronic pain.^1^ They stated that chronic pain severity that could not be explained by tissue abnormality was often doubted with patients accused of “symptom magnification, symptom embellishment, amplified pain behaviour, exaggerated symptoms or disability seeking behaviours and malingering.” At that time, as it is now, there was no validated objective test that could measure the subjective experience of chronic pain. Although various tests and procedures had been used to identify exaggeration in patients with traumarelated brain injury, tests of feigned mental disorders have not been sufficiently validated in the setting of chronic pain. In that same series, Mendelson and Mendelson stated that malingering (the willful, deliberate, and fraudulent feigning or exaggeration of symptoms of illness or injury, done for the purpose of a consciously desired end) is not a medical or psychiatric diagnosis and the determination of a person malingering is a legal finding.^2^ Now 20 years on, with better understanding of the complex neurophysiological mechanisms underlying chronic pain, but still without a biomarker, we encounter the same dilemma of validating patient self-report of pain severity and functional impairment especially when there is contention.

This current paper explores widespread misunderstandings and misconceptions surrounding chronic pain within independent medical evaluations (IMEs), particularly when assessments are conducted by assessors trained primarily in musculoskeletal (MSK) disorders and with insufficient knowledge of the science of pain medicine. Reliance on objective physical findings to motivate a medical opinion does not align with the complex nature of chronic pain. We aim to highlight that chronic pain is frequently misinterpreted by healthcare professionals causing patients to be wrongly labeled as exaggerating symptoms and leading to disadvantage and stigmatization – especially in medicolegal contexts. These misperceptions risk biasing the assessor’s conclusions and, ultimately, the adjudication of claims.

## Chronic pain as a contentious condition

Chronic pain is not exclusive to a single diagnosis and represents a multifactorial condition that transcends the traditional biomedical model. It is the leading cause of disability worldwide and it is often more complex than specific MSK disorders. Chronic pain is often erroneously attributed to mental illness but is not inherently a psychiatric condition. Chronic pain is referenced in the Diagnostic and Statistical Manual of Mental disorders, Fifth Edition (DSM5) as a component of somatic symptom disorder (SSD).^3^ This diagnostic category does indicate that the patient is sufficiently distressed by the somatic symptom of pain that it creates psychological distress, and in turn, the psychological distress may increase the perception of pain. Thus, the diagnosis of somatic symptom disorder with predominant pain indicates that pain is not viewed as a standalone symptom but occurs in the context of a psychological response to the symptom. Emphasizing a combination of physical and mental health symptoms should lead to helpful treatment recommendations. Katz et al. have, however, argued that SSD is an overly inclusive and unreliable disorder with the risk that medical illness including chronic pain conditions will be misdiagnosed/misinterpreted as mental illness.^4^ Healthcare practitioner education in chronic pain is generally lacking with risk that medicolegal evaluators may be poorly informed and continue to base evaluations on outdated information. This educational gap leads to poor understanding of the complex nature of chronic pain, with a tendency to view pain as a symptom rather than a standalone disease – consequences that are amplified in legal settings.

Even with the current knowledge of disordered neurophysiological mechanisms underlying chronic pain, the absence of biomarkers or consistent objective findings causes symptom validity to be doubted and raises issues of dishonest amplification. These assumptions set the stage for an IME assessor, without sufficient pain medicine knowledge, to be biased at the outset. In such situations, valid impairments may be unconsciously underrecognized, not due to ill intent, but due to knowledge gaps and biases toward more familiar disease models. The impression of dishonest symptom amplification is particularly relevant when there is alleged functional impact that interferes with family, social and work activities. Those in the pain medicine community are more cognizant of these challenges, especially with the greater understanding of nociplastic pain, a condition that cannot be sufficiently explained by tissue abnormality. In contrast, the courts or adjudicating bodies are traditionally heavily reliant on objective parameters to aid in reaching a decision and are less willing to accept that a seemingly nebulous condition can cause symptoms sufficiently severe to impact function. Current scientific concepts such as central sensitization or maladaptive neuroplasticity remain underrecognized or dismissed in legal proceedings. This leads to the premise that patients with chronic pain may be exaggerating symptoms for secondary gain, with the risk that those who are truly suffering will be unfairly judged.

The belief that persons with chronic pain commonly misrepresent their symptoms is illustrated by a recent systematic review (SR) on prevalence of symptom exaggeration among North American IME examinees by Darzi and colleagues.^5^ These authors report that almost 50% of women and 33% of men undergoing IMEs for chronic pain or traumatic brain injury are exaggerating symptoms. We acknowledge that among patients with chronic pain there will be those that are truly suffering, some will exaggerate symptoms (for reasons that may be personal, socioeconomically related, or for secondary gain) and there will always be a small group who are truly malingering.^6^ In a review of chronic pain disease simulation, Fishbain et al. reported that malingering may occur in up to 10% of patients, but with inconsistent prevalence within patient groups and with absence of a reliable method for detecting malingering.^7^ Furthermore, any claims that malingering can be determined by specific testing should be viewed with caution. But to continue to promote the concept of important symptom exaggeration by many will disadvantage patients, perpetuate the stigma, and serve the interests of insurers, employers, among others.

## The independent medical evaluation

As a start, the IME process must be understood. An IME is requested when there is contention regarding a health issue, which could be the diagnosis, causation of a condition, or functional ability. An IME encounter is an artificial situation for many reasons. The traditional doctor–patient relationship is replaced by a healthcare professional who is employed to make a medical assessment, often based only on a single encounter, and the patient is not identified as such, but rather as a claimant, plaintiff, or a defendant. The IME assessor must apply clinical knowledge and expertise, together with a sound understanding of the current science to formulate an opinion that will assist the trier of fact in reaching a decision. Different from the treating clinician, the assessor is not an advocate for the patient and should not pander to the wants of the requesting party. Above all, the assessor must remain objective and unbiased. Once there is resort to a legal context and when the usual health record is called into question, patients may feel compromised, with those in lower socioeconomic or minoritized groups more likely to be disproportionately affected. There may be the concern that the treating healthcare professional has not provided sufficient or accurate information to satisfy the adjudicating body, or those with an alternative motive to obtain secondary gain may be dissatisfied that the medical records do not support their claim. Either way, the IME experience is different from the usual healthcare encounter, has the potential to disrupt trusting healthcare relationships and for many may be a cause for distress.

Considerable gaps exist between current research in chronic pain conditions and medicolegal standards. Tools commonly used to assess chronic pain such as pain scales and psychosocial questionnaires, commonly used in clinical practice or research settings, lack formal validation for use in the medicolegal context. Neurocognitive testing, used to detect symptom exaggeration or malingering of mental abilities, has been suggested as a measure of pain exaggeration.^8^ These tests have not been sufficiently tested or validated in chronic pain populations and cannot be used as a surrogate to identify exaggeration of pain or functional status. These tests simply tell us that a claimant responded in a manner that reflects symptom exaggeration ***on a particular test***. The result does not negate disability, as any opinion regarding a degree of symptom exaggeration that might reflect malingering would require multiple points of convergent data, including clinical interview, review of health records and examination. This emphasizes that a clinician must be highly diligent and pursue all avenues prior to relying too heavily on one piece of evidence reflective of exaggeration.

## Malingering in the context of medicolegal evaluation of chronic pain

Malingering is neither a medical nor psychiatric diagnosis, but rather a complex and multifaceted phenomenon that cannot be determined by intuition or isolated behavioral cues. Simplistic or premature judgments based on observations of inconsistent behavior, mechanical testing such as range of motion or strength of exertion or response to various questionnaires are fraught with bias. Behavioral changes or inconsistencies – often interpreted as deception – can occur for reasons, such as distress, miscommunication, or cultural factors.Symptom inconsistencies or amplification may stem from nonvolitional mechanisms such as trauma-related hypervigilance, coexisting psychiatric conditions, sociocultural influences, or fear of invalidation. To determine whether symptom inconsistencies are conscious (i.e., malingering) or unconscious (e.g., somatization, catastrophizing, or maladaptive coping) remains highly challenging – particularly in chronic pain conditions that are poorly understood, lack objective biomarkers, and are increasingly classified as nociplastic pain, for which validated assessment tools are still absent. In this context, labeling a patient’s symptoms as “exaggeration” or “malingering” without clear and consistent evidence is both scientifically unfounded and ethically inappropriate, risking the delegitimization of patient experiences and reinforcing stigma, especially among those already navigating diagnostic and care barriers. Although the current literature often uses exaggeration and malingering synonymously, we contend that this is erroneous for reasons stated above. Exaggeration may occur as a subconscious element in the presentation of chronic pain with influence by biomedical and psychosocial factors. Exaggeration and functional impairment or disablement are not mutually exclusive.

Physical techniques of isokinetic testing, such as for Waddell signs or Jamar dynamometer to measure grip strength, have been challenged as an indicator of submaximal effort or malingering, and could not reliably discriminate between submaximal or best effort.^7^ Neurocognitive tests have also been erroneously used to diagnose malingering in chronic pain patients.^9^ Intentional cognitive dysfunction does not equate with pain simulation.^10^ Neurocognitive impairment is increasing recognized to occur in patients with chronic pain either due to the pain experience, medication side effects or the associated features of poor sleep, fatigue and mood disturbance.^11^ However, at this time neurocognitive findings cannot be used as a surrogate for assessment of level of chronic pain, amount of physical impairment or evidence for exaggeration/malingering of chronic pain. Criteria for identifying malingering in chronic pain patients were proposed by Bianchini and colleagues and were an adaptation of a neuropsychological questionnaire to detect malingered cognitive deficits in the context of traumatic brain injury.^8,12^ These proposed criteria fall short of current understanding of neurophysiological mechanisms underlying chronic pain and do not align with contemporary pain science. Examples of fallacious content in these criteria include effort bias (e.g., Jamar grip test), reference to nonorganic findings such as Waddell’s sign, reliance on collateral informants (e.g., friends or relatives), presence of cognitive changes in the absence of head injury, self-reported symptoms as discrepant with known patterns of physiological or neurological functioning (e.g., whole body pain in a patient with a small right-sided cervical disc bulge), among others.^8^ Various tests of cognition may suggest a link between malingered cognitive dysfunction and exaggerated pain but cannot be used as a surrogate for pain exaggeration or malingering.^9,13,14^

In the DSM-5 “malingering” is identified as a condition requiring further study and appears under “Other Conditions That May Be a Focus of Clinical Attention.”^3^ Numerous authors have offered a precise and medicolegally sound definition of malingering – as the conscious intent to deceive for external gain.^1,2^ They emphasize that this is a legal, not medical classification, reinforcing the role of the forensic assessor as an objective evaluator rather than an arbiter of motive.

## The role of a medicolegal assessor

The role of the medicolegal assessor is not to judge motive but to present the facts by making observations, collecting, analyzing, and interpreting clinical findings objectively. The assessor may make comment on various inconsistencies in the history or examination of a claimant, as well as poor treatment compliance, poor motivation and if applicable the presence of a defined psychiatric disorder but should not overinterpret “nonorganic” signs. Determining the veracity of a claimant is the prerogative of the courts.

It is essential that the assessor is impartial with the credibility of the report reliant on neutrality. An assessor is not allowed to pronounce on “malingering,” which falls outside the scope of a clinical evaluation. Most evaluations in the context of chronic pain center on function, physical findings, and pain behavior, but do not encompass the forensic expertise required to assess deception. When exaggeration/malingering is suspected – particularly when it may involve cognitive or behavioral manipulation – a formal neuropsychological or forensic psychiatric assessment is more appropriate.

The concept of intentional deception must be distinguished from secondary gain. Chronic pain patients may emphasize their suffering to validate their experience – not to deceive. This phenomenon, sometimes referred to as validation distress, reflects the psychological toll of repeatedly feeling disbelieved. These expressions are rooted in the desire for recognition, not malice. Conflating such behavior with malingering reflects a misunderstanding of the pain experience. Therefore, determining whether a claimant is malingering involves attributing intent, which falls within the purview of legal – not medical – authorities. The courts, insurers, and administrative tribunals are ultimately responsible for making judgments about credibility and motive.

## Recent publication concerning medicolegal evaluation of chronic pain patients

A cursory reading of the SR reporting exaggeration by chronic pain patients in IME settings will leave the reader with the impression of highly prevalent “exaggeration” by persons with chronic pain.^5^ On closer scrutiny, we believe this report to be misleading and raises questions of accuracy and validity. Symptom exaggeration across 44 studies (9,794 individuals attending IMEs) with 31 studies of traumatic brain injury (TBI) and 11 of chronic pain, was reported to range from 17% to 67%, and to occur in almost 50% of women and in approximately 33% of men.^5^ This conclusion was based on pooling of all studies, chronic pain and TBI patients, diverse conditions with vastly different effects on global functioning.

Review of the 11 studies cited for chronic pain reveals that almost all participants reported spinal pain (mostly low back pain) following an injury. Other pain comorbid conditions, when reported, comprised less than 10%. Although low back pain is the most common cause for chronic pain, focus almost entirely on post traumatic spinal pain misrepresents a large proportion of patients with other causes of chronic pain who may be referred for an IME. Secondly, patients were further preselected by being referred for exhaustive and costly neuropsychological testing which is not the norm for the majority of patients with chronic pain referred for an IME. This referral pattern may reflect the distinct focus of this group of psychologists practicing in southeastern United States and should not be applied to a more diverse population of patients referred for IMEs in another jurisdiction.

Although Darzi uses the terminology “exaggeration,” 10 of the studies included reported “malingering” based on criteria proposed by Bianchini et al., criteria which we assess as fallacious,^8^ and one study was based entirely on neuropsychological parameters.^12^ With included studies reported from 2006 to 2019, reported malingering increased from 40% in 2006 to over 80% (13.7% assessed as not malingered pain related disability) in 2019. It is curious that the criteria used to identify malingering in the 11 studies quoted, were developed in 2005 by the same authors reporting on the 11 studies and have not been sufficiently validated in other settings.

Furthermore, we believe that the numbers of subjects reported is inaccurate for the following reasons: all studies were reported by the same authors; almost all patients were identified retrospectively from archival records of persons referred for “psychological evaluations at a clinical psychology practice in the southeastern United States” in the context of mostly disability claims; the sum total of chronic pain participants is over 4200 with time frames overlapping and extending from 1998 to 2012 when reported; in only two studies was there acknowledgment that 40% of 318 and 11.3% of 612 participants had previously been reported in two of the 11 previous studies^15,16^; in two studies reported in 2010 (612 participants) and 2014 (328 participants), the patient recruitment strategy is identical “Clinical patients were culled from the archival records of 772 sequential cases … from 1998 through 2003” and “clinical cases were obtained from the records of a series of 772 referrals for psychological pain evaluation at a clinical psychology practice … from 1998 through 2003.”^16,17^ Therefore, there is considerable doubt that each study reported on different patients with the concern that information on the same patient was repeatedly reported in different studies causing the number of over 4000 to be greatly inflated.

Therefore, for these many reasons we contend that the conclusion of excessive symptom exaggeration for chronic pain patients as reported in this SR are suspect and may not reflect the reality of patients with chronic pain referred for IMEs.

## Concluding thoughts

Chronic pain medicolegal evaluations require an in-depth exploration of biopsychosocial factors, often involving narrative data, contextual variables, and nonphysical evidence and are inherently time consuming. Patients with chronic pain are at risk of being stigmatized based on allegations of exaggeration or malingering. Symptom exaggeration and disability are, however, not mutually exclusive, with some truly disabled individuals presenting exaggerated symptoms or findings on clinical examination. Equating chronic pain with mental health conditions is erroneous, especially in the IME setting. Persons with mental health symptoms are distinctly different from those with chronic pain and any testing to identify symptom exaggeration in mental health conditions cannot be unequivocally applied to other illnesses. We have concerns that current dogma may unfairly bias clinicians in their assessment of validity of pain report and functional ability in those with chronic pain.
